# Intraoperative 40 Hz gamma frequency auditory stimulation for postoperative sleep disturbance in patients undergoing laparoscopic gynecological surgery: protocol for a randomized controlled trial

**DOI:** 10.3389/fmed.2026.1813371

**Published:** 2026-04-16

**Authors:** Xiaoxuan Hu, Chong Fu, Binyang Ding, Yaoyu Ying, Qiyun Tan, Wei-ming Zhao, Yajuan Zhu, Tingting Wu, Juan Wang, Hong Liu, Lingzhong Meng, Fu-hai Ji, Hua-yue Liu

**Affiliations:** 1 Department of Anesthesiology, First Affiliated Hospital of Soochow University, Suzhou, Jiangsu, China; 2 Division of Life Sciences and Medicine, School of Biomedical and Engineering, University of Science and Technology of China, Hefei, China; 3 Department of Epidemiology and Biostatistics, School of Public Health, Suzhou Medical College of Soochow University, Suzhou, China; 4 Department of Obstetrics and Gynecology, First Affiliated Hospital of Soochow University, Suzhou, Jiangsu, China; 5 Department of Anesthesiology and Pain Medicine, University of California Davis Health, Sacramento, CA, United States; 6 Department of Anesthesia, Indiana University School of Medicine, Indianapolis, IN, United States; 7 Ambulatory Surgery Center, The First Affiliated Hospital of Soochow University, Suzhou, Jiangsu, China

**Keywords:** 40 Hz auditory stimulation, laparoscopic gynecologic surgery, perioperative medicine, postoperative sleep disturbance, randomized controlled trial

## Abstract

**Background:**

Postoperative sleep disturbance (PSD) is a common complication after surgery and is associated with impaired recovery. This protocol describes a randomized controlled trial designed to determine whether intraoperative 40 Hz gamma frequency auditory stimulation reduces the incidence of PSD after laparoscopic gynecological surgery.

**Methods:**

This randomized, double-blind, controlled trial will enroll 342 patients scheduled for laparoscopic gynecological surgery under general anesthesia. Patients will be randomly allocated in a 1:1:1 ratio to three parallel groups: control (no headphones), sham stimulation (headphones without auditory output), or active stimulation (headphones delivering 40 Hz auditory stimulation). The primary outcome is the incidence of PSD on the first postoperative night. Secondary outcomes include the incidence of PSD on postoperative nights 2 and 3, daily Athens Insomnia Scale (AIS) scores, anxiety and depression scores (HADS-A/-D), sedative-hypnotic use, pain scores, analgesic consumption, the incidence of postoperative nausea and vomiting (PONV), rescue antiemetic use, duration of post-anesthesia care unit (PACU) stay, length of postoperative hospital stay, quality of postoperative recovery (QoR-15), perioperative adverse events, and patient satisfaction.

**Discussion:**

This trial will evaluate whether intraoperative 40 Hz gamma frequency auditory stimulation reduces PSD in patients undergoing laparoscopic gynecological surgery. The findings may provide evidence for a non-invasive perioperative strategy to mitigate sleep disruption and improve postoperative recovery.

**Clinical trial registration:**

http://www.chictr.org.cn, identifier [ChiCTR2500110341].

## Introduction

Postoperative sleep disturbance (PSD) is a common complication after surgery under general anesthesia and is characterized by difficulty initiating sleep, frequent nocturnal awakenings, and poor sleep quality (1). The reported incidence ranges from 15% to 72% among surgical patients (2–5). Beyond compromising patient comfort, PSD is associated with a higher risk of postoperative delirium and delayed recovery (6, 7). Contributing factors include pain, anxiety, comorbidities, anesthetic technique, surgical trauma, and perioperative medication exposure (3, 6). Although minimally invasive techniques, particularly laparoscopy, are now predominant in gynecological surgery (8), the incidence of PSD remains substantial due to individual variability, pneumoperitoneum-related effects, and perioperative medication use. A systematic review indicates that non-pharmacologic interventions such as physical sleep aids, relaxation techniques, and music therapy can improve sleep quality in hospitalized patients (9). However, individual variability in response to these measures underscores the need for evidence-based, easily implementable protocols that optimize perioperative sleep quality.

Sensory stimulation at 40 Hz (auditory or light) can entrain gamma-frequency neural oscillations, promote coordinated network activity, facilitate glymphatic clearance of metabolic waste, and attenuate disease progression in Alzheimer’s disease (AD) (10, 11). Notably, daily combined visual and auditory 40 Hz stimulation administered for more than 6 months improved sleep quality in patients with AD (12). Gamma oscillations (30–80 Hz) subserve memory consolidation, sensory perception, attention, information processing, and neuroprotection (13, 14). Despite these advances in neurodegenerative disease, no study has examined whether 40 Hz auditory stimulation can attenuate PSD in the surgical setting.

This randomized controlled trial aims to evaluate whether intraoperative 40 Hz gamma-frequency auditory stimulation reduces the incidence of PSD in patients undergoing laparoscopic gynecological surgery under general anesthesia. The findings would establish a novel, non-invasive strategy to improve perioperative sleep quality and facilitate postoperative recovery.

## Methods

### Ethics and registration

Ethics approval was obtained from the Institutional Review Board of the First Affiliated Hospital of Soochow University on 30 September 2025 (approval No. 2025-919). The trial was registered with the Chinese Clinical Trial Registry on 13 October 2025 (http://www.chictr.org.cn/showproj.html?proj=290309; identifier: ChiCTR2500110341). This protocol adheres to the Standard Protocol Items: Recommendations for Interventional Trials (SPIRIT) 2025 statement (Supplementary Table 1) (15). This trial will be conducted in accordance with the Declaration of Helsinki, and written informed consent will be obtained from all patients prior to enrollment. This manuscript represents Protocol Version 1.0, dated 24 September 2025.

### Study design and status

This single-center, randomized, double-blind, controlled clinical trial will enroll 342 patients at the First Affiliated Hospital of Soochow University, Suzhou, China. Following anesthesia induction, patients scheduled for laparoscopic gynecological surgery will be randomly allocated in a 1:1:1 ratio to one of three parallel groups: Group A (control, no headphones), Group B (sham, wearing headphones without auditory stimulation), or Group C (intervention, wearing headphones with 40 Hz gamma frequency auditory stimulation delivered for 60 min intraoperatively).

**FIGURE 1:**
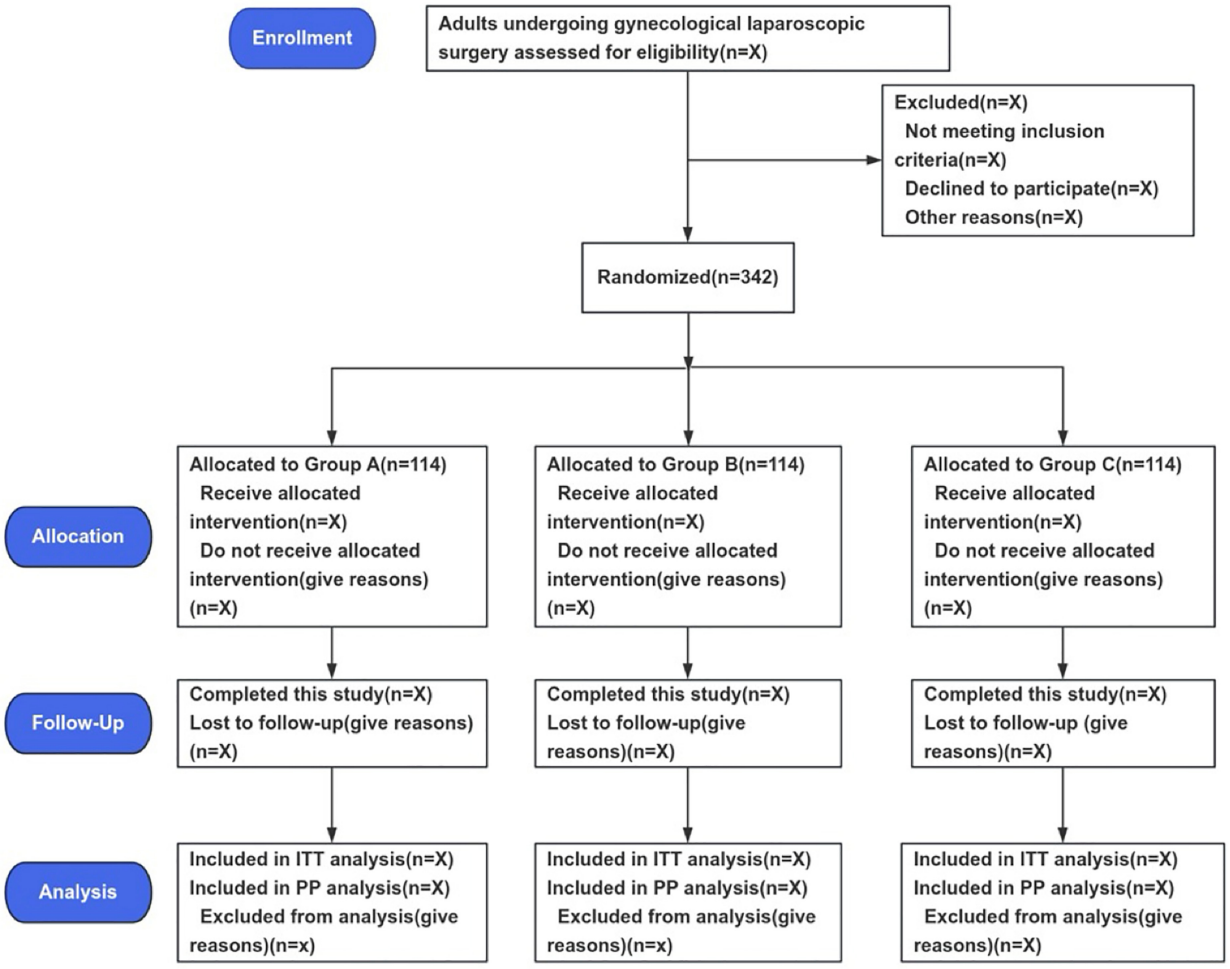
Study flowchart.

This trial started on October 14, 2025, and the anticipated completion date is 31 December 2026. At the time this manuscript was prepared, participant enrollment had already begun; however, all follow-up data will be recorded and maintained in an electronic data management platform and will remain concealed until completion of the final statistical analysis. Figure 1 presents the study flow diagram, and Table 1 outlines the schedule of enrollment, interventions, and outcome assessments according to the SPIRIT statement.

**TABLE 1:** Schedule of patient enrollment, study interventions, and measurements complying with the Standard Protocol Items: Recommendations for Interventional Trials (SPIRIT) statement.

Time point	Study period							
	Enrollment	Allocation	Post-allocation					
	Preoperative visit	Before surgery	During surgery	PACU	Day 1 postoperatively	Day 2 postoperatively	Day 3 postoperatively	Hospital discharge
Enrollment	–	–	–	–	–	–	–	–
Inclusion criteria	×	–	–	–	–	–	–	–
Exclusion criteria	×	–	–	–	–	–	–	–
Written informed	×	–	–	–	–	–	–	–
Baseline characteristics	×	–	–	–	–	–	–	–
PSQI scores^a^	×	–	–	–	–	–	–	–
AIS scores^b^	×	–	–	–	–	–	–	–
HADS-A scores^c^	×	–	–	–	–	–	–	–
HADS-D scores^d^	×	–	–	–	–	–	–	–
Apfel scores^e^	×	–	–	–	–	–	–	–
Preoperative comorbidities	×	–	–	–	–	–	–	–
Oral sedative-hypnotics	×	–	–	–	–	–	–	–
Randomization	–	×	–	–	–	–	–	–
Allocation	–	×	–	–	–	–	–	–
Interventions	–	–	–	–	–	–	–	–
Group A	–	×	–	–	–	–	–	–
Group B	–	×	–	–	–	–	–	–
Group C		×	–	–	–	–	–	–
Endpoints measures	–	–	–	–	–	–	–	–
AIS scores	–	–	–	–	×	×	×	–
The use of sedative-hypnotics	–	–	–	–	×	×	×	–
HADS-A scores	–	–	–	–	×	×	×	–
HADS-D scores	–	–	–	–	×	×	×	–
Incidence of PONV	–	–	–	×	×	×	×	–
Antiemetic rescue	–	–	–	×	×	×	×	–
NRS scores at rest^f^	–	–	–		×	×	×	–
NRS scores while coughing^f^	–	–	–	–	×	×	×	–
The use of rescue analgesics	–	–	–	–	×	×	×	–
Length of PACU stay	–	–	–	×	–	–	–	–
Length of hospital stay	–	–	–	–	–	–	–	×
Perioperative anaesthesia-related adverse events^g^	–	–	–	–	–	–	–	×
Medication use for adverse event management	–	–	–	–	–	–	–	×
QoR-15 scores^h^	–	–	–	–	×	×	×	–
Patient satisfaction	–	–	–	–	×	×	×	–
Time of return to ward	–	–	–	–	×	–	–	–
Night time care frequency	–	–	–	–	×	×	×	–

According to Standard Protocol Items: Recommendations for Interventional Trials (SPIRIT) statement of defining standard protocol items for clinical trials.

*^a^*PSQI scores: Pittsburgh Sleep Quality Index scores.

*^b^*AIS scores: Athens Insomnia Scale scores.

*^c^*HADS-A scores: Hospital Anxiety and Depression Scale Anxiety subscale.

*^d^*HADS-D scores: Hospital Anxiety and Depression Scale Depression subscale.

*^e^*Apfel scores: PONV risk scoring system, the risk factors include female sex, non-smoking status, history of motion sickness or PONV, and postoperative opioid use.

*^f^*NRS: numeric rating scale.

*^g^*Perioperative anaesthesia-related adverse events: including: hypertension, hypotension, bradycardia, tachycardia, desaturation, postoperative shivering, emergence agitation, allergic reactions, severe arrhythmias.

*^h^*QoR-15 scores: Quality of Recovery 15 scores.

### Participants and enrollment

The inclusion criteria are as follows: (1) female patients aged 18 years or older who are scheduled to undergo laparoscopic gynecological surgery under general anesthesia; and (2) an expected operative duration (from induction of anesthesia to surgical closure) of at least 60 min.

The exclusion criteria are as follows: (1) pregnancy, lactation, or current menstruation; (2) long-term use (>3 months) of antidepressants or psychotropic medications, or substance abuse within the previous 6 months; (3) comorbidities including: severe respiratory insufficiency or uncontrolled bronchial asthma, uncontrolled hypertension (systolic blood pressure > 180 mmHg or diastolic blood pressure > 110 mmHg despite medication), abnormal liver or kidney function (Child-Pugh class C or receipt of renal replacement therapy), or severe cardiovascular diseases (New York Heart Association class III–IV heart failure, myocardial infarction within the previous 6 months, or unstable angina); (4) a history of epilepsy or seizure disorders; (5) pre-existing neurological disorders (e.g., stroke, dementia, or Parkinson’s disease), psychiatric conditions requiring hospitalization, or inability to communicate because of language barriers or cognitive impairment; (6) a physical inability to wear headphones (e.g., auricular deformity or external auditory canal abnormality); (7) participation in another interventional clinical trial within 4 weeks before enrollment; and (8) known hypersensitivity or contraindications to anesthetic agents or adjuvant medications used in the study protocol.

The withdrawal criteria are as follows: (1) withdrawal of informed consent at any time or an explicit participant request to discontinue study participation; (2) occurrence of a serious adverse event necessitating investigator-initiated discontinuation for safety reasons; and (3) any other circumstance that precludes continued participation (e.g., major protocol violation, intercurrent illness, or loss to follow-up).

The termination criteria are as follows: (1) premature compromise of blinding; (2) identification of major protocol deficiencies that threaten scientific validity; and (3) protracted delays in participant enrollment or frequent major protocol deviations.

### Randomization and blinding

This study will employ a stratified block randomization design. An independent researcher who is not involved in patient recruitment, data collection and management, or statistical analysis will generate the randomization sequence using an online randomization tool.^1^ Randomization will be performed in a 1:1:1 ratio with randomly varying block sizes of 3 and 6 and will be stratified by Pittsburgh Sleep Quality Index (PSQI) score assessed one month preoperatively (PSQI ≤ 5 vs. > 5). The allocation sequence will be concealed in sequentially numbered, opaque, sealed envelopes.

In the preoperative holding area, an anesthesia nurse who is not involved in patient recruitment, intraoperative anesthesia management, or postoperative outcome assessment will fit each patient with headphones and adjust the volume to the minimum audible level while the patient is resting with her eyes closed. The headphones will then be removed. After induction of anesthesia, the same anesthesia nurse will open the envelope in sequence and implement the assigned intervention for Group A (control group), Group B (sham group), and Group C (intervention group).

To maintain blinding, each patient’s head will be covered with an opaque surgical towel after induction so that the presence or absence of headphones is not visible to the surgical team. Patients, surgeons, nursing staff, and anesthesia providers managing the case will be blinded to group allocation. Postoperative outcome assessors and the statistician performing the final analysis will remain blinded to treatment assignment throughout the study. Participants will be informed of their group allocation after completion of all follow-up assessments. Emergency unblinding will be permitted only when essential for patient safety; all such events will be documented and reported to the institutional review board. A post-procedure blinding integrity assessment will also be performed: within 24 h after full recovery from anesthesia, each participant will complete a standardized questionnaire asking her to guess her group allocation (control, sham, or intervention) and to rate her confidence in that guess (certain, somewhat certain, or guessing). Outcome assessors will independently record their allocation guess after completing the day-3 assessment.

### Study interventions

Patients in Group A (control) will not wear headphones. Patients in Group B (sham) will wear headphones without 40 Hz gamma frequency auditory stimulation. Patients in Group C (intervention) will wear headphones delivering 40 Hz gamma frequency auditory stimulation for 60 min, after which the headphone will remain in place without further stimulation until the end of surgery. The headphone model will be Sennheiser MOMENTUM 4 (Sonova Consumer Hearing GmbH; speaker frequency response range, 6 Hz–22 KHz). The 40 Hz gamma frequency auditory stimulus will be generated using MATLAB 2022b (MathWorks Inc., United States) at a sampling rate of 44,100 Hz and stored as an uncompressed WAV file (16-bit PCM). The stimulus will be delivered bilaterally at 60 dB sound pressure level, which is below the occupational exposure limit of 85 dB, via Sennheiser MOMENTUM 4 headphones, and output intensity will be verified using a Type 2 sound level meter (IEC 61672). The MATLAB source code and generated WAV file are provided as Supplementary File 2. To verify stimulus delivery, protocol adherence will be defined as receipt of ≥95% of the prescribed 60-min stimulation duration; any deviation will be documented in the case report form.

### Perioperative management

Participants will be admitted to standardized wards (≤2 patients per room, lights out at 20:00, and ambient noise ≤ 45 dB from 20:00 to 06:00) and will undergo preoperative fasting for at least 6 h for solids and 2 h for clear liquids in accordance with current guidelines. Upon arrival in the operating room, standard monitoring will include continuous electrocardiography, peripheral oxygen saturation (SpO_2_), non-invasive blood pressure, and the Bispectral Index (BIS, Aspect Medical Systems, Newton, Massachusetts, United States) (16).

Anesthesia will be induced with intravenous sufentanil 0.2–0.4 μg/kg, ciprofol 0.4 mg/kg (17) and lidocaine 1.5 mg/kg administered sequentially. When the Modified Observer’s Alertness/Sedation Scale (MOAA/S) score reaches 0, rocuronium 0.6 mg/kg will be administered to facilitate endotracheal intubation. Anesthesia will be maintained with sevoflurane (approximately 1.5%–2.5%, titrated to maintain a BIS of 40–60), supplemented with intermittent boluses of sufentanil 0.1–0.2 μg/kg and a continuous infusion of remifentanil 0.1–0.2 μg/kg/min, adjusted according to surgical stimulation and hemodynamic response. Mechanical ventilation will target a tidal volume of 6–8 mL/kg predicted body weight, an end-tidal CO2 of 35–45 mmHg, and a positive end-expiratory pressure (PEEP) of 5–10 cmH2O. Normothermia (36 °C–37 °C) will be maintained with forced-air warming. Additional rocuronium will be administered as needed to maintain adequate neuromuscular blockade. At the end of surgery, neuromuscular blockade will be reversed with sugammadex 200 mg. Extubation will proceed once consciousness has returned, commands can be followed, and spontaneous ventilation is adequate. PACU discharge will require a modified Aldrete score ≥ 9.

A multimodal analgesic approach will be used, consisting of ultrasound-guided bilateral posterior rectus sheath blocks or transversus abdominis plane (TAP) blocks performed after induction of anesthesia and before surgical incision, using 20 mL of 0.375% ropivacaine per side (total dose, 150 mg) (18), together with intravenous flurbiprofen axetil 50 mg administered at the end of surgery. Postoperative pain will be assessed using the Numerical Rating Scale (NRS; 0–10, where 0 indicating no pain and 10 indicating the most severe pain) (19). Rescue analgesia with intravenous tramadol 50 mg will be administered for an NRS score ≥ 4 at rest. A two-drug PONV prophylaxis regimen will be implemented, consisting of intravenous dexamethasone 8 mg immediately after induction of anesthesia and intravenous ondansetron 8 mg [a selective 5-hydroxytryptamine 3 (5-HT3) receptor antagonist] at the end of surgery.

Intraoperatively and in the PACU, hemodynamic and respiratory events will be managed according to protocol as follows: hypertension (systolic blood pressure ≥ 160 mmHg or 20% above baseline) with intravenous nicardipine 0.2 mg; hypotension (systolic blood pressure ≤ 90 mmHg or 20% below baseline) with intravenous ephedrine 6 mg or phenylephrine 50–100 μg; bradycardia (heart rate < 50 beats/min sustained for ≥ 1 min) with intravenous atropine 0.25–0.5 mg; tachycardia (heart rate > 100 beats/min sustained for ≥ 1 min) with intravenous esmolol 20 mg; and desaturation (SpO_2_ < 92%) with supplemental oxygen at 5–10 L/min via nasal cannula or mask ventilation.

### Perioperative assessments

An independent research coordinator who is not involved in patient care or outcome assessment will record baseline data, including age, menopausal status, body mass index, ASA physical status, smoking and alcohol history, comorbidities, current medications (particularly sedative-hypnotic, antidepressants, and anxiolytics), vital signs, and relevant laboratory values.

The Pittsburgh Sleep Quality Index (PSQI; 7 components: subjective sleep quality, sleep latency, sleep duration, sleep efficiency, use of sleep medications, sleep disturbances, and daytime dysfunction; each scored 0–3, total score 0–21; scores > 5 indicating poor sleep quality) (20) will be administered to assess sleep quality during the preceding month. Preoperative AIS and HADS scores will also be assessed. The simplified Apfel risk score for PONV (female sex, non-smoking status, prior motion sickness or PONV, and anticipated postoperative opioid use; range: 0–4) (21) will be calculated for each participant.

Intraoperative data will include anesthetic agents and doses, surgery type (e.g., adnexal surgery, hysterectomy, or other gynecological procedures) and indication (benign or malignant), duration of anesthesia (from induction to emergence), duration of surgery (from incision to closure), intraoperative fluid balance (crystalloids, colloids, blood products, estimated blood loss, and urine output), and adverse events. Postoperative data will include time of arrival on the ward from the PACU, frequency of nighttime nursing interventions (defined as staff entries into the patient room between 20:00 and 06:00) on postoperative nights 1–3, pathology results, and all prespecified outcome measures.

### Data monitoring committee

Data will be recorded on standardized case report form and entered into an electronic database with access restricted to authorized study personnel. A standing Data Monitoring Committee (DMC), composed of senior anesthesiology clinicians and qualified statisticians, has been convened to adjudicate any disputes or uncertainties arising during data acquisition and to assess whether postoperative analgesic agents administered to enrolled participants fall outside the boundaries of the approved protocol. The committee includes a chairperson with extensive anesthesiology experience, one attending anesthesiologist, two statisticians, and one attending thoracic surgeon. In the event of discrepancies in recorded data or questions regarding protocol-concordant medication use, these issues will be formally referred to the DMC for structured review and a definitive determination.

### Trial outcome definitions

#### Primary outcome

The primary outcome is the incidence of PSD on the first postoperative night, defined as Athens Insomnia Scale (AIS) scores ≥ 6, consistent with clinically meaningful sleep impairment. The AIS comprises eight items: night awakenings, difficulty falling sleep, early final awakening, total sleep duration, overall sleep quality, sense of wellbeing, daytime functioning, and daytime sleepiness (22).

#### Secondary outcomes

Secondary outcomes, each assessed on postoperative days 1–3 unless otherwise specified, are as follows: (1) the incidence of PSD on postoperative nights 2 and 3; (2) AIS scores; (3) Hospital Anxiety and Depression Scale anxiety and depression subscale scores (HADS-A, HADS-D; 7 items each scored 0–3, with subscale scores ≥ 8 indicating clinically significant symptoms) (2, 23); (4) sedative-hypnotic use on postoperative nights 1–3; (5) pain scores at rest and during coughing (NRS) and analgesic consumption; (6) the incidence of PONV and rescue antiemetic use in the PACU and on postoperative days 1–3; (7) duration of PACU stay; (8) postoperative length of hospital stay; (9) quality of recovery assessed by the QoR-15 (15 items scored 0–10, maximum 150; higher scores indicate superior recovery) (24); (10) perioperative adverse events; and (11) patient satisfaction assessed using a five-point Likert scale (5 = highly satisfied, 4 = satisfied, 3 = neutral, 2 = dissatisfied, and 1 = very dissatisfied).

#### Safety outcomes

Safety assessment will include intraoperative and postoperative adverse events for each participant. Adverse events comprise hypertension, hypotension, bradycardia, tachycardia, oxygen desaturation, postoperative shivering, emergence agitation, allergic reactions, hypothermia, severe ventricular arrhythmias, and cardiac arrest. Detailed definitions are provided in Supplementary File 1. All adverse events will be documented during anesthesia, in the PACU, and on the surgical ward. Any serious adverse event related to the study intervention must be reported to the Data Monitoring Committee (DMC) within 24 h using a standardized adverse event form. A serious adverse event may necessitate unblinding, protocol modification, or participant withdrawal.

#### Sample size calculation

Sample size estimation was based on a pilot study conducted from August to September 2025 that enrolled 25 patients per group undergoing laparoscopic gynecological surgery under general anesthesia. The observed incidence of PSD on the first postoperative night was 44% (11/25) in the control group, 40% (10/25) in the sham stimulation group, and 24% (6/25) in the intervention group, consistent with published rates of 15%–72% after abdominal surgery (2–5). On the basis of these data, we assumed PSD incidences of 45%, 40%, and 20% in the control, sham, and intervention groups, respectively. For a three-group comparison with a two-sided significance level of α = 0.025 (Bonferroni-adjusted for two pairwise comparisons: intervention vs. control and intervention vs. sham) and 80% power, the required sample size was 98 per group, as calculated using R software (version 4.5.1^2^). Accounting for the asymptotic relative efficiency of the Mann-Whitney U test relative to the *t*-test (0.955) under potential non-normally, and anticipating a 10% dropout rate, the total sample size was determined to be 342 patients (114 per group).

### Statistical analysis

The primary analysis will follow the intention-to-treat (ITT) principle, and a per-protocol (PP) analysis will be performed as a sensitivity analysis. The ITT population will comprise all randomized patients who undergo surgery and receive at least a partial intervention. Exclusion criteria for the PP analysis are as follows: (1) sedative-hypnotic use within 72 h postoperatively; (2) patient-controlled intravenous analgesia (PCIA) containing opioids or sedative-hypnotic; (3) intravenous rather than sevoflurane-based maintenance; (4) conversion to open laparotomy; and (5) use of non-protocol agents (e.g., dexmedetomidine, esketamine, or benzodiazepines).

Baseline characteristics will be summarized using descriptive statistics and compared across groups using the absolute standardized difference (ASD) to assess randomization balance. An ASD > 0.1 will indicate meaningful imbalance requiring adjustment in the primary analysis. For continuous variables, normality will be assessed using the Shapiro-Wilk test. Normally distributed data will be reported as mean ± standard deviation (SD) and compared using the *t*-test; non-normally distributed data will be reported as median (interquartile range, IQR) and compared using the Mann-Whitney U test. Categorical variables will be presented as counts (percentages) and compared using the Chi-squared test or Fisher’s exact test, as appropriate.

The primary outcome will be compared across the three groups using the chi-square test, followed by pairwise comparisons (intervention vs. control and intervention vs. sham) using log-binomial regression, with results reported as risk ratios (RRs), risk differences (RD), and 95% confidence intervals (CIs). The Bonferroni correction will maintain the family-wise error rate at *P* < 0.025 for each comparison. Covariates with ASD > 0.1 will be incorporated into multivariable log-binomial models to estimate adjusted risk ratios and adjusted risk differences with 95% CI. Secondary outcomes will be compared among groups using methods appropriate to the data distribution: independent-samples *t*-test or Mann-Whitney U test for continuous variables, and chi-square test or Fisher’s exact test for categorical variables. To control for multiple comparisons, the Benjamini-Hochberg false discovery rate (FDR) procedure will be applied using a threshold of q < 0.025. Treatment effects for continuous outcomes will be reported as mean differences (MD) with 95% CIs, and categorical outcomes as RRs, RDs, and 95% CI. For outcomes assessed on postoperative days 1 through 3 (AIS, HADS-A, HADS-D, NRS, QoR-15), a linear mixed-effects model with fixed effects for treatment group, postoperative day, and their interaction, together with a random intercept for each participant, will serve as the primary longitudinal analysis. Repeated binary outcomes (e.g., daily PSD incidence) will be analyzed using generalized estimating equations with a logit link and an exchangeable working correlation structure.

Prespecified subgroup analyses for the primary outcomes will be conducted according to age (18–64 years vs. ≥ 65 years), menopausal status (premenopausal *vs.* postmenopausal), postoperative sedative-hypnotics use (yes vs. no), postoperative pain intensity (NRS < 4 vs. ≥ 4), postoperative analgesic use (yes vs. no), PONV occurrence (yes vs. no), postoperative HADS-A scores (≥8 vs. < 8), postoperative HADS-D scores (≥8 vs. < 8), duration of surgery (<2 vs. ≥2 h), time of return to the ward (before 20:00 vs. after 20:00), and pathological diagnosis (benign vs. malignant). Subgroup effects will be assessed by testing interaction tests.

All statistical tests will be two-sided, with *P* < 0.025 considered statistically significant. No interim analyses will be performed. The primary analysis will use a complete-case approach; however, if the proportion of missing outcome data exceeds 5%, multiple imputation will be performed, and the imputed results will be reported alongside the primary analysis. All analyses will be conducted using R software (version 4.5.1, see text footnote 2). A detailed statistical analysis plan has been prepared and is provided as Supplementary File 1.

## Discussion

This protocol describes a single-center, randomized, double-blind, controlled trial evaluating whether intraoperative 40 Hz gamma frequency auditory stimulation reduces PSD in 342 adults undergoing laparoscopic gynecological surgery. The primary objective is to determine whether this intervention reduces the incidence of PSD on the first postoperative night compared with control and sham conditions. Secondary objectives include PSD on postoperative nights 2 and 3, the incidence of PONV and rescue antiemetic use, duration of PACU and hospital stay, postoperative pain scores, analgesic consumption, quality of recovery, and patient satisfaction. The trial will be conducted and reported in accordance with the Consolidated Standards of Reporting Trials (CONSORT) 2025 statement (25).

Sensory stimulation at 40 Hz is an emerging non-invasive neuromodulation approach. Multimodal 40 Hz stimulation has reduced emergence delirium in children, suppressed epileptiform activity, and conferred neuroprotective effects in preclinical studies (26–28). Although non-invasive brain stimulation techniques such as transcranial alternating current stimulation and transcranial magnetic stimulation, can attenuate postoperative sleep disturbances (29, 30), these modalities require specialized equipment and trained personnel, which limits their scalability in routine perioperative care. In contrast, auditory 40 Hz stimulation offers a cost-effective alternative that can be readily integrated into standard anesthetic practice.

Whether such stimulation can engage neural circuits under general anesthesia remains an important consideration. The 40 Hz auditory steady-state response (ASSR) is attenuated but not abolished during sevoflurane anesthesia, retaining approximately 21% of its awake amplitude (31). Moreover, the neuroprotective actions of 40 Hz stimulation extend beyond cortical entrainment: preclinical studies have demonstrated modulation of microglial phenotype, attenuation of neuroinflammation, and enhanced glymphatic clearance (32, 33), processes mediated in part by subcortical auditory pathways that remain relatively intact under volatile anesthesia. Although cortical ASSRs are substantially suppressed at a BIS of 40–60, subcortical processing and neuroimmune modulation may mediate therapeutic effects independently of cortical oscillatory synchronization. Fu et al. (26) provided direct clinical evidence supporting this premise by demonstrating measurable neurobiological effects of 40 Hz sensory stimulation during sevoflurane anesthesia. Available evidence also supports the safety of gamma-frequency stimulation. A systematic review found no increase in adverse events compared with sham stimulation (34). Similarly, the 6-month OVERTURE trial reported no treatment-related serious adverse events or seizures (35), and Chan et al. observed no adverse events during 2 years of daily 40 Hz audiovisual stimulation (36).

Several limitations warrant acknowledgment. First, enrollment is restricted to patients undergoing laparoscopic gynecological surgery, which may limit the generalizability of the findings to other surgical populations, particularly older adults undergoing major surgery or non-laparoscopic procedures. Second, as a single-center study, the external validity of the findings will need to be confirmed through multicenter replication. Third, the trial addresses acute, surgery-related sleep disturbance; whether 40 Hz auditory stimulation is beneficial for chronic insomnia, or which parameters (frequency, duration, timing) are most effective for mitigating PSD, requires separate investigation. Fourth, the mechanistic pathways linking 40 Hz auditory stimulation to perioperative sleep regulation remain incompletely characterized; further basic science research is needed to elucidate the neurobiological mechanisms underlying any observed effects on PSD.

Despite these limitations, this trial addresses a clinically significant problem. If effective, intraoperative 40 Hz auditory stimulation would provide a safe, non-invasive, and scalable intervention to improve postoperative sleep quality. Conversely, a null finding would still refine current understanding of neuromodulation strategies for PSD prevention and guide the design of future trials exploring alternative stimulation parameters or delivery modalities. Regardless of outcome, this research will advance knowledge regarding the role of intraoperative sensory interventions in optimizing perioperative neurocognitive health.

## Footnotes

1
https://www.sealedenvelope.com/simple-randomiser/v1/lists

2
http://www.R-project.org

## References

[1] Niklasson A Finan P Smith M Forsberg A Dietz N Kander T et al. The relationship between preoperative sleep disturbance and acute postoperative pain control: a systematic review and meta-analysis. *Sleep Med Rev.* (2025) 79:102014. 10.1016/j.smrv.2024.102014 39504912

[2] Qiu D Wang X Yang J Chen S Yue C Hashimoto K et al. Effect of intraoperative esketamine infusion on postoperative sleep disturbance after gynecological laparoscopy: a randomized clinical trial. *JAMA Netw Open.* (2022) 5:e2244514. 10.1001/jamanetworkopen.2022.44514 36454569 PMC9716381

[3] Su X Wang D. Improve postoperative sleep: what can we do? *Curr Opin Anaesthesiol.* (2018) 31:83–8. 10.1097/ACO.0000000000000538 29120927 PMC5768217

[4] Chouchou F Khoury S Chauny J Denis R Lavigne G. Postoperative sleep disruptions: a potential catalyst of acute pain? *Sleep Med Rev.* (2014) 18:273–82. 10.1016/j.smrv.2013.07.002 24074687

[5] Gögenur I Wildschiøtz G Rosenberg J. Circadian distribution of sleep phases after major abdominal surgery. *Br J Anaesth.* (2008) 100:45–9. 10.1093/bja/aem340 18037670

[6] Butris N Tang E Pivetta B He D Saripella A Yan E et al. The prevalence and risk factors of sleep disturbances in surgical patients: a systematic review and meta-analysis. *Sleep Med Rev.* (2023) 69:101786. 10.1016/j.smrv.2023.101786 37121133

[7] O’Gara B Gao L Marcantonio E Subramaniam B. Sleep, pain, and cognition: modifiable targets for optimal perioperative brain health. *Anesthesiology.* (2021) 135:1132–52. 10.1097/ALN.0000000000004046 34731233 PMC8578455

[8] Boza A Urman B Vatansever D Ceyhan M Mısırlıoglu S Koca S et al. Mini-laparoscopic gynecological surgery using smaller ports minimizes incisional pain and postoperative scar size: a paired sample analysis. *Surg Innov.* (2020) 27:455–60. 10.1177/1553350620923526 32501743

[9] Beswick A Wylde V Bertram W Whale K. The effectiveness of non-pharmacological sleep interventions for improving inpatient sleep in hospital: a systematic review and meta-analysis. *Sleep Med.* (2023) 107:243–67. 10.1016/j.sleep.2023.05.004 37257367

[10] Murdock M Yang C Sun N Pao P Blanco-Duque C Kahn M et al. Multisensory gamma stimulation promotes glymphatic clearance of amyloid. *Nature.* (2024) 627:149–56. 10.1038/s41586-024-07132-6 38418876 PMC10917684

[11] Zhou X He Y Xu T Wu Z Guo W Xu X et al. 40 Hz light flickering promotes sleep through cortical adenosine signaling. *Cell Res.* (2024) 34:214–31. 10.1038/s41422-023-00920-1 38332199 PMC10907382

[12] Cimenser A Hempel E Travers T Strozewski N Martin K Malchano Z et al. Sensory-evoked 40-Hz gamma oscillation improves sleep and daily living activities in Alzheimer’s disease patients. *Front Syst Neurosci.* (2021) 15:746859. 10.3389/fnsys.2021.746859 34630050 PMC8500065

[13] Mulkey M Albanese T Kim S Huang H Yang B. Delirium detection using GAMMA wave and machine learning: a pilot study. *Res Nurs Health.* (2022) 45:652–63. 10.1002/nur.22268 36321335 PMC9649882

[14] Etter G van der Veldt S Manseau F Zarrinkoub I Trillaud-Doppia E Williams S. Optogenetic gamma stimulation rescues memory impairments in an Alzheimer’s disease mouse model. *Nat Commun.* (2019) 10:5322. 10.1038/s41467-019-13260-9 31757962 PMC6876640

[15] Chan A Boutron I Hopewell S Moher D Schulz K Collins G et al. SPIRIT 2025 statement: updated guideline for protocols of randomised trials. *BMJ.* (2025) 389:e081477. 10.1136/bmj-2024-081477 40294953 PMC12035670

[16] Struys M Vanpeteghem C Huiku M Uutela K Blyaert N Mortier E. Changes in a surgical stress index in response to standardized pain stimuli during propofol-remifentanil infusion. *Br J Anaesth.* (2007) 99:359–67. 10.1093/bja/aem173 17609248

[17] Gan T Bertoch T Habib A Yan P Zhou R Lai Y et al. Comparison of the Efficacy of HSK3486 and propofol for induction of general anesthesia in adults: a multicenter, randomized, double-blind, controlled, phase 3 noninferiority trial. *Anesthesiology.* (2024) 140:690–700. 10.1097/ALN.0000000000004886 38150544

[18] Liu T Tuo J Wei Q Sun X Zhao H Zhao X et al. Effects of abdominal wall blocks on postoperative delirium in elderly patients undergoing laparoscopic surgery: a randomized controlled study. *Med Sci Monit.* (2022) 28:e934281. 10.12659/MSM.934281 35283476 PMC8932049

[19] Breivik H Borchgrevink P Allen S Rosseland L Romundstad L Hals E et al. Assessment of pain. *Br J Anaesth.* (2008) 101:17–24. 10.1093/bja/aen103 18487245

[20] Buysse D Reynolds C Monk T Berman S Kupfer D. The pittsburgh sleep quality index: a new instrument for psychiatric practice and research. *Psychiatry Res.* (1989) 28:193–213. 10.1016/0165-1781(89)90047-4 2748771

[21] Gan T Diemunsch P Habib A Kovac A Kranke P Meyer T et al. Consensus guidelines for the management of postoperative nausea and vomiting. *Anesth Analg.* (2014) 118:85–113. 10.1213/ANE.0000000000000002 24356162

[22] Soldatos C Dikeos D Paparrigopoulos T. Athens insomnia scale: validation of an instrument based on ICD-10 criteria. *J Psychosom Res.* (2000) 48:555–60. 10.1016/s0022-3999(00)00095-7 11033374

[23] Fomenko A Dümmler D Aktürk Z Eck S Teusen C Karapetyan S et al. Hospital anxiety and depression scale anxiety subscale (HADS-A) for detecting anxiety disorders in adults. *Cochrane Database Syst Rev.* (2025) 7:CD015456. 10.1002/14651858.CD015456 40600405 PMC12216811

[24] Stark P Myles P Burke J. Development and psychometric evaluation of a postoperative quality of recovery score: the QoR-15. *Anesthesiology.* (2013) 118:1332–40. 10.1097/ALN.0b013e318289b84b 23411725

[25] Hopewell S Chan A Collins G Hróbjartsson A Moher D Schulz K et al. CONSORT 2025 statement: updated guideline for reporting randomised trials. *BMJ.* (2025) 389:e081123. 10.1136/bmj-2024-081123 40228833 PMC11995449

[26] Fu S Bian J Fu Y Yang Y Wang Y Zhu G et al. 40-Hz light stimulation and emergence delirium incidence after sevoflurane Anesthesia in children: a randomized clinical trial. *JAMA Pediatr.* (2025) 179:1300–7. 10.1001/jamapediatrics.2025.3903 41082242 PMC12519408

[27] Wang C Lin C Zhao Y Samantzis M Sedlak P Sah P et al. 40-Hz optogenetic stimulation rescues functional synaptic plasticity after stroke. *Cell Rep.* (2023) 42:113475. 10.1016/j.celrep.2023.113475 37979173

[28] Wang L Xu W Wang K Yang J Li H Wang Q et al. Chronic 40 Hz light flicker mitigates epileptogenesis through a visual pathway associated with the dorsal lateral geniculate nucleus shell. *Nat Commun.* (2025) 16:9228. 10.1038/s41467-025-64269-2 41107227 PMC12534601

[29] Meier J Nolte G Schneider T Engel A Leicht G Mulert C. Intrinsic 40Hz-phase asymmetries predict tACS effects during conscious auditory perception. *PLoS One.* (2019) 14:e0213996. 10.1371/journal.pone.0213996 30943251 PMC6447177

[30] Du N Yang J Li X Li Z Hashimoto K Lei L et al. Prophylactic effect of prefrontal alternating current stimulation on postoperative sleep disturbance in patients undergoing gynecological laparoscope: a randomized, double-blind, sham-controlled trial. *CNS Neurosci Ther.* (2025) 31:e70529. 10.1111/cns.70529 40755300 PMC12320130

[31] Plourde G Chartrand D Fiset P Font S Backman S. Antagonism of sevoflurane anaesthesia by physostigmine: effects on the auditory steady-state response and bispectral index. *Br J Anaesth.* (2003) 91:583–6. 10.1093/bja/aeg209 14504163

[32] Adaikkan C Middleton S Marco A Pao P Mathys H Kim D et al. Gamma entrainment binds higher-order brain regions and offers neuroprotection. *Neuron.* (2019) 102: 929–43.e8. 10.1016/j.neuron.2019.04.011 31076275 PMC6697125

[33] Martorell A Paulson A Suk H Abdurrob F Drummond G Guan W et al. Multi-sensory gamma stimulation ameliorates Alzheimer’s-associated pathology and improves cognition. *Cell.* (2019) 177:256–71.e22. 10.1016/j.cell.2019.02.014 30879788 PMC6774262

[34] Wu L Wei Y He K Gao Q. The effects and safety of gamma rhythm stimulation on cognitive function in Alzheimer’s disease: a systematic review and meta-analysis. *Neurorehabil Neural Repair.* (2025) 39:1046–59. 10.1177/15459683251360733 40855942

[35] Hajós M Boasso A Hempel E Shpokayte M Konisky A Seshagiri C et al. Safety, tolerability, and efficacy estimate of evoked gamma oscillation in mild to moderate Alzheimer’s disease. *Front Neurol.* (2024) 15:1343588. 10.3389/fneur.2024.1343588 38515445 PMC10957179

[36] Chan D de Weck G Jackson B Suk H Milman N Kitchener E et al. Gamma sensory stimulation in mild Alzheimer’s dementia: an open-label extension study. *Alzheimers Dement.* (2025) 21:e70792. 10.1002/alz.70792 41137616 PMC12552893

