# Supplementary material for: Intraoperative 40 Hz gamma frequency auditory stimulation for postoperative sleep disturbance in patients undergoing laparoscopic gynecological surgery: protocol for a randomized controlled trial
Source: Front Med (Lausanne). 2026 Apr 16;13:1813371. doi: 10.3389/fmed.2026.1813371 (PMC13128588; doi:10.3389/fmed.2026.1813371)
Supplement: Supplementary file 2 [file Supplementary_file_1.docx]

**Statistical Analysis Plan (SAP)**

**Intraoperative 40 Hz Gamma Frequency Auditory Stimulation for**

**Postoperative Sleep Disturbance in Patients Undergoing**

**Laparoscopic Gynecological Surgery: A Randomized Controlled Trial**

*Chinese Clinical Trial Registry identifier: ChiCTR2500110341*

SAP Version 1.0

Date: September 2025

# 1. Administrative Information

| **Role** | **Name** | **Affiliation** | **Date** |
| --- | --- | --- | --- |
| Author / Trial Statistician | Xiaoxuan Hu, MS | Department of Anesthesiology, First Affiliated Hospital of Soochow University | 2026-03 |
| Chief Investigator | Hua-yue Liu, MD, PhD | Department of Anesthesiology, First Affiliated Hospital of Soochow University | 2026-03 |
| Independent Statistician | Yao-yu Ying | Department of Epidemiology and Biostatistics, School of Public Health, Soochow University | 2026-03 |

# 2. Introduction

This document details the statistical analysis plan for the randomized controlled trial evaluating the effect of intraoperative 40 Hz gamma frequency auditory stimulation on postoperative sleep disturbance (PSD) in patients undergoing laparoscopic gynecological surgery. The clinical trial is registered at the Chinese Clinical Trial Registry (ChiCTR2500110341). This SAP should be read in conjunction with the trial protocol (Version 1.0, dated 24 September 2025).

## 2.1 Background and Rationale

Postoperative sleep disturbance (PSD) is a common complication after surgery under general anesthesia, characterized by difficulty initiating sleep, frequent nocturnal awakenings, and poor sleep quality. The reported incidence ranges from 15% to 72% among surgical patients. Beyond compromising patient comfort, PSD is associated with a higher risk of postoperative delirium and delayed recovery.

Sensory stimulation at 40 Hz (auditory or light) can entrain gamma-frequency neural oscillations, promote coordinated network activity, facilitate glymphatic clearance of metabolic waste, and attenuate disease progression in Alzheimer’s disease. Daily combined visual and auditory 40 Hz stimulation for over six months improved sleep quality in AD patients. Despite these advances, no study has examined whether 40 Hz auditory stimulation can attenuate PSD in the surgical setting.

This trial investigates whether intraoperative 40 Hz gamma frequency auditory stimulation can reduce the incidence of PSD in patients undergoing laparoscopic gynecological surgery under general anesthesia.

## 2.2 Objectives

The primary objective is to determine whether intraoperative 40 Hz gamma frequency auditory stimulation reduces the incidence of PSD on the first postoperative night (defined as Athens Insomnia Scale [AIS] score ≥ 6) compared to control and sham conditions.

Secondary objectives include: (1) To compare PSD incidence on postoperative nights 2 and 3; (2) To assess the effect on daily AIS scores across postoperative days 1–3; (3) To evaluate anxiety and depression scores (HADS-A/D); (4) To assess sedative-hypnotic use, pain scores, and analgesic consumption; (5) To evaluate PONV incidence and rescue antiemetic use; (6) To assess duration of PACU stay and postoperative hospital length of stay; (7) To evaluate quality of recovery (QoR-15); (8) To assess perioperative adverse events; and (9) To evaluate patient satisfaction.

# 3. Study Methods

## 3.1 Trial Design

This is an investigator-initiated, single-center, prospective, parallel-group, randomized, double-blind, controlled clinical trial. Participants are randomized in a 1:1:1 ratio to one of three parallel groups: Group A (control group, no headphones), Group B (sham group, wearing headphones without auditory stimulation), or Group C (intervention group, wearing headphones delivering 40 Hz gamma frequency auditory stimulation for 60 minutes intraoperatively).

The three-arm design was adopted to disentangle the specific effect of 40 Hz auditory stimulation from any nonspecific effects of headphone wearing (e.g., ambient noise attenuation, pressure sensation, or placebo expectation). The comparison between Group A and Group B isolates the contribution of headphone wearing per se, while the comparison between Group B and Group C isolates the specific effect of 40 Hz auditory stimulation.

## 3.2 Randomization

A stratified block randomization design is employed, with randomization stratified by Pittsburgh Sleep Quality Index (PSQI) score assessed one month preoperatively (PSQI ≤ 5 vs. > 5). Randomization is conducted in a 1:1:1 allocation ratio using randomly varying block sizes of 3 and 6. An independent researcher, not involved in patient recruitment, data collection, data management, or statistical analysis, generates the randomization sequence using an online randomization tool (sealedenvelope.com). The randomization sequence is concealed in sequentially numbered, opaque, sealed envelopes.

## 3.3 Blinding

To maintain blinding, each patient’s head is covered with an opaque surgical towel after anesthesia induction so that the presence or absence of headphones is not visible to the surgical team. Patients, surgeons, nurses, and anesthesia providers managing the case are blinded to group allocation. Postoperative outcome assessors and the statistician performing the final analysis remain blinded to treatment assignment throughout the study. A post-procedure blinding integrity assessment using the James blinding index will be performed.

## 3.4 Power and Sample Size

Sample size estimation was based on a pilot study (August–September 2025, n = 25 per group). The observed PSD incidence on the first postoperative night was 44% (11/25) in the control group, 40% (10/25) in the sham group, and 24% (6/25) in the intervention group. Assumed PSD incidences are 45%, 40%, and 20% in the control, sham, and intervention groups, respectively. For a three-group comparison with a two-sided significance level of α = 0.025 (Bonferroni-adjusted for two pairwise comparisons: intervention vs. control and intervention vs. sham) and 80% power, the required sample size was 98 per group. Accounting for the asymptotic relative efficiency of the Mann–Whitney U test compared to the t-test (0.955) for potentially non-normally distributed data, and anticipating a 10% dropout rate, the total sample size is 342 patients (114 per group). Sample size was calculated using R software (version 4.5.1).

# 4. Statistical Principles

## 4.1 Confidence Intervals and P-values

All estimates of differences between groups will be presented with two-sided 95% confidence intervals. The primary analysis uses a Bonferroni-corrected significance threshold of P < 0.025 per pairwise comparison (intervention vs. control; intervention vs. sham) to maintain the family-wise error rate at 5%.

## 4.2 Modeling Principles

For the primary binary outcome, pairwise comparisons will be conducted using log-binomial regression to estimate risk ratios (RR) and risk differences (RD) with 95% confidence intervals. If the log-binomial model fails to converge, modified Poisson regression with a log link and robust (Huber–White sandwich) standard errors will be used as an alternative. For continuous outcomes assessed repeatedly over postoperative days 1–3 (AIS, HADS-A, HADS-D, NRS, QoR-15), a linear mixed-effects model (LMM) with fixed effects for treatment group, time (postoperative day), and the group × time interaction, and a random intercept for each participant, will be used. For repeated binary outcomes (e.g., daily PSD incidence), generalized estimating equations (GEE) with a logit link and exchangeable correlation structure will be applied.

## 4.3 Multiple Comparisons

The primary outcome will be tested at the Bonferroni-corrected threshold of P < 0.025 per pairwise comparison. For secondary outcomes, the Benjamini–Hochberg false discovery rate (FDR) procedure will be applied with a threshold of q < 0.025. Secondary outcomes should be considered exploratory given the risk of type I error from multiple comparisons.

## 4.4 Analysis Sets

### 4.4.1 Intention-to-Treat (ITT) Population

The ITT population comprises all randomized patients who undergo surgery and receive at least partial intervention. The primary analysis will adhere to the ITT principle. Patients are analyzed according to their randomized group assignment.

### 4.4.2 Per-Protocol (PP) Population

The PP population includes patients adhering strictly to the study protocol. PP exclusion criteria include: (1) sedative-hypnotic use within 72 hours postoperatively; (2) patient-controlled intravenous analgesia (PCIA) containing opioids or sedative-hypnotics; (3) intravenous rather than sevoflurane-based maintenance; (4) conversion to open laparotomy; and (5) use of non-protocol agents (e.g., dexmedetomidine, esketamine, or benzodiazepines). The PP analysis will serve as a sensitivity analysis.

## 4.5 Missing Data

The proportion of missing data for the primary and secondary outcomes will be reported. The primary analysis will be conducted using available data (complete-case analysis). Should the proportion of missing primary or secondary outcome data exceed 5%, sensitivity analyses will be conducted using multiple imputation by chained equations (MICE) under the missing-at-random (MAR) assumption with 20 imputed datasets, as well as best-case/worst-case scenario analyses, to assess the robustness of the primary conclusion. Linear mixed-effects models used for repeated-measures outcomes inherently accommodate incomplete observations under the MAR assumption.

# 5. Definition and Derived Variables

## 5.1 Primary Outcome

The primary outcome is the incidence of postoperative sleep disturbance (PSD) on the first postoperative night, defined as Athens Insomnia Scale (AIS) score ≥ 6. The AIS comprises eight items: night awakenings, difficulty falling asleep, early final awakening, total sleep duration, perceived sleep quality, sense of well-being, daytime functioning, and daytime sleepiness. Each item is scored 0–3, yielding a total score of 0–24, with scores ≥ 6 indicating clinically meaningful sleep impairment.

## 5.2 Secondary Outcomes

| **Outcome** | **Definition** | **Data Type** | **Values / Coding** |
| --- | --- | --- | --- |
| PSD on nights 2–3 | AIS ≥ 6 on postoperative nights 2 and 3 | Binary | 1 = Yes, 0 = No |
| AIS scores | Athens Insomnia Scale total score (days 1–3) | Continuous | 0–24 (higher = worse) |
| HADS-A scores | Hospital Anxiety and Depression Scale – Anxiety subscale | Continuous | 0–21; ≥ 8 = clinically significant |
| HADS-D scores | Hospital Anxiety and Depression Scale – Depression subscale | Continuous | 0–21; ≥ 8 = clinically significant |
| Sedative-hypnotic use | Use of sedative-hypnotics on postoperative nights 1–3 | Binary | 1 = Yes, 0 = No |
| NRS at rest | Numerical Rating Scale pain score at rest (days 1–3) | Continuous | 0–10 (0 = no pain, 10 = worst) |
| NRS with coughing | NRS pain score during coughing (days 1–3) | Continuous | 0–10 |
| Analgesic consumption | Use of rescue analgesics (tramadol) days 1–3 | Binary | 1 = Yes, 0 = No |
| PONV incidence | Occurrence of nausea/vomiting in PACU and days 1–3 | Binary | 1 = Yes, 0 = No |
| Rescue antiemetic use | Need for rescue antiemetics in PACU and days 1–3 | Binary | 1 = Yes, 0 = No |
| Duration of PACU stay | Time from PACU admission to discharge (Aldrete ≥ 9) | Continuous | Minutes |
| Hospital length of stay | Duration of postoperative hospital stay | Continuous | Days |
| QoR-15 scores | Quality of Recovery-15 (days 1–3) | Continuous | 0–150 (higher = better) |
| Patient satisfaction | 5-point Likert scale on days 1–3 | Ordinal | 1 = very dissatisfied to 5 = highly satisfied |

## 5.3 Safety Outcomes

| **Safety Outcome** | **Definition** | **Data Type** |
| --- | --- | --- |
| Hypertension | Systolic BP ≥ 160 mmHg or ≥ 20% above baseline | Binary |
| Hypotension | Systolic BP ≤ 90 mmHg or ≥ 20% below baseline | Binary |
| Bradycardia | Heart rate < 50 bpm sustained for ≥ 1 min | Binary |
| Tachycardia | Heart rate > 100 bpm sustained for ≥ 1 min | Binary |
| Oxygen desaturation | SpO₂ < 92% (PACU: sustained >15 s on 3 L/min O₂ nasal cannula) | Binary |
| Postoperative shivering | Visible shivering in PACU | Binary |
| Emergence agitation | Agitation during emergence from anesthesia | Binary |
| Allergic reactions | Any allergic reaction to study medications | Binary |
| Hypothermia | Core temperature < 36°C | Binary |
| Severe ventricular arrhythmias | Clinically significant ventricular arrhythmia | Binary |
| Cardiac arrest | Cardiac arrest requiring intervention | Binary |
| Any adverse event | Composite of any above adverse events | Binary |

## 5.4 Subgroup Variables

| **Subgroup Variable** | **Definition** | **Categories** |
| --- | --- | --- |
| Age | Patient age at enrollment | 18–64 years vs. ≥ 65 years |
| Menopausal status | Self-reported menopausal status | Premenopausal vs. postmenopausal |
| Sedative-hypnotic use | Postoperative sedative-hypnotic use | Yes vs. No |
| Pain intensity | Postoperative NRS score | NRS < 4 vs. NRS ≥ 4 |
| Analgesic use | Postoperative rescue analgesic use | Yes vs. No |
| PONV occurrence | Postoperative nausea and vomiting | Yes vs. No |
| HADS-A scores | Postoperative anxiety subscale | ≥ 8 vs. < 8 |
| HADS-D scores | Postoperative depression subscale | ≥ 8 vs. < 8 |
| Surgery duration | Duration from incision to closure | < 2 hours vs. ≥ 2 hours |
| Time of return to ward | Time of ward arrival after PACU | Before 20:00 vs. after 20:00 |
| Pathological diagnosis | Final pathology result | Benign vs. malignant |

# 6. Trial Population

## 6.1 Recruitment

A CONSORT flow diagram will be produced to describe participant flow through each stage of the trial, including the numbers screened, randomized, allocated to each of the three groups, followed up, and analyzed. Reasons for exclusion and loss to follow-up will be documented.

## 6.2 Baseline Characteristics

Categorical data will be summarized by frequencies and percentages. Continuous data will be summarized by means and standard deviations if normally distributed (assessed by Shapiro–Wilk test), or medians and interquartile ranges if skewed.

Baseline balance between groups will be assessed using the absolute standardized difference (ASD). ASD > 0.1 indicates meaningful imbalance requiring adjustment in the primary analysis. Where baseline P-values are presented in tables, they will be considered descriptive only.

Baseline characteristics to be summarized include: age, menopausal status, body mass index, ASA physical status classification, smoking and alcohol history, comorbidities, current medications (sedative-hypnotics, antidepressants, anxiolytics), vital signs, laboratory values, PSQI scores, preoperative AIS scores, HADS-A and HADS-D scores, and Apfel simplified risk scores for PONV.

# 7. Statistical Analysis Methods

## 7.1 Primary Outcome Analysis

**Outcome:** Incidence of PSD on the first postoperative night (AIS ≥ 6), binary.

**Unadjusted Analysis:** Overall comparison across three groups using the chi-squared test. Pairwise comparisons (intervention vs. control; intervention vs. sham) using log-binomial regression, reporting risk ratios (RR), risk differences (RD), and 95% confidence intervals (CI). The Bonferroni correction maintains the family-wise error rate at P < 0.025 per comparison.

**Adjusted Analysis:** Covariates with ASD > 0.1 will be incorporated into multivariable log-binomial models, yielding adjusted risk ratios (aRR) and adjusted risk differences (RD) with 95% CI. If the log-binomial model fails to converge, modified Poisson regression with robust standard errors will be used.

**Covariates:** Any baseline variables with ASD > 0.1 (potential covariates include age, BMI, ASA status, PSQI score, baseline AIS score, Apfel score, surgery type).

**Effect Measures:** Adjusted RR (95% CI) and adjusted RD (95% CI) as the primary effect measures; unadjusted estimates will be reported for transparency.

**Sensitivity Analysis:** (1) Repeat analysis using PP population; (2) If missing data > 5%, multiple imputation (20 datasets, MAR) and best-case/worst-case scenario analyses.

## 7.2 Secondary Outcome Analysis – Detailed Methods by Outcome

| **Outcome** | **Time Points** | **Data Type** | **Unadjusted Test** | **Adjusted Analysis** | **Effect Measure** |
| --- | --- | --- | --- | --- | --- |
| PSD nights 2–3 | Days 2–3 | Binary | Chi-squared or Fisher’s exact | GEE (logit link, exchangeable) | aRR (95% CI), q-value |
| AIS scores | Days 1–3 | Continuous | Mann–Whitney U | LMM (group × time + random intercept) | MD (95% CI), q-value |
| HADS-A scores | Days 1–3 | Continuous | Mann–Whitney U | LMM (group × time + random intercept) | MD (95% CI), q-value |
| HADS-D scores | Days 1–3 | Continuous | Mann–Whitney U | LMM (group × time + random intercept) | MD (95% CI), q-value |
| Sedative-hypnotic use | Nights 1–3 | Binary | Chi-squared or Fisher’s exact | GEE (logit link) | aRR (95% CI), q-value |
| NRS at rest | Days 1–3 | Continuous | Mann–Whitney U | LMM (group × time + random intercept) | MD (95% CI), q-value |
| NRS with coughing | Days 1–3 | Continuous | Mann–Whitney U | LMM (group × time + random intercept) | MD (95% CI), q-value |
| Analgesic consumption | Days 1–3 | Binary | Chi-squared or Fisher’s exact | GEE (logit link) | aRR (95% CI), q-value |
| PONV incidence | PACU, Days 1–3 | Binary | Chi-squared or Fisher’s exact | GEE (logit link) | aRR (95% CI), q-value |
| Rescue antiemetic use | PACU, Days 1–3 | Binary | Chi-squared or Fisher’s exact | GEE (logit link) | aRR (95% CI), q-value |
| Duration of PACU stay | Post-surgery | Continuous | Mann–Whitney U | Linear regression | MD (95% CI) |
| Hospital length of stay | Discharge | Continuous | Mann–Whitney U | Linear regression | MD (95% CI) |
| QoR-15 scores | Days 1–3 | Continuous | Mann–Whitney U | LMM (group × time + random intercept) | MD (95% CI), q-value |
| Patient satisfaction | Days 1–3 | Ordinal | Kruskal–Wallis | Ordinal logistic regression | OR (95% CI), q-value |

## 7.3 Pairwise Comparison Strategy

For all outcome comparisons, the following pairwise comparisons will be performed: (1) Intervention (Group C) vs. Control (Group A); (2) Intervention (Group C) vs. Sham (Group B). The comparison between Control (Group A) and Sham (Group B) will be reported descriptively but is not a primary or secondary hypothesis test.

## 7.4 Longitudinal Analysis – Linear Mixed-Effects Models

## For outcomes assessed repeatedly on postoperative days 1, 2, and 3 (AIS, HADS-A, HADS-D, NRS, and QoR-15), linear mixed-effects models (LMMs) will be employed as the primary longitudinal analysis. Each model will include fixed effects for treatment group (three levels), time (postoperative day, treated as a categorical variable), and the group × time interaction, along with a random intercept for each participant to account for within-subject correlation across repeated measurements.

## This modeling framework offers several advantages. First, it naturally accommodates incomplete observations under the missing-at-random (MAR) assumption without requiring complete cases, thereby maximizing the use of available data. Second, the inclusion of the group × time interaction term permits estimation of time-specific treatment effects, enabling detection of differential intervention effects across postoperative days. These effects will be estimated using pre-specified contrasts derived from the interaction model, with pairwise comparisons (intervention vs. control; intervention vs. sham) evaluated at each time point.

## Model covariance structure selection will proceed as follows: an unstructured covariance matrix will be compared against the default random-intercept specification using Akaike Information Criterion (AIC) and Bayesian Information Criterion (BIC), and the structure yielding the better fit will be adopted. If model convergence issues arise with the unstructured specification, a compound symmetry or autoregressive (AR-1) structure will be considered as alternatives. Residual diagnostics, including normality of residuals and homoscedasticity, will be examined graphically. Where distributional assumptions are substantially violated, robust standard errors or non-parametric alternatives will be applied as sensitivity analyses.

## 7.5 Safety Outcome Analysis

| **Safety Outcome** | **Statistical Test** | **Effect Measure** | **Notes** |
| --- | --- | --- | --- |
| Hypertension | Fisher’s exact test | RR (95% CI), RD | Expected low frequency |
| Hypotension | Fisher’s exact test | RR (95% CI), RD | Expected low frequency |
| Bradycardia | Fisher’s exact test | RR (95% CI), RD | Expected low frequency |
| Tachycardia | Fisher’s exact test | RR (95% CI), RD | Expected low frequency |
| Oxygen desaturation | Fisher’s exact test | RR (95% CI), RD | Expected low frequency |
| Postoperative shivering | Fisher’s exact test | RR (95% CI), RD | Expected low frequency |
| Emergence agitation | Fisher’s exact test | RR (95% CI), RD | Expected low frequency |
| Allergic reactions | Fisher’s exact test | RR (95% CI), RD | Expected rare |
| Hypothermia | Fisher’s exact test | RR (95% CI), RD | Expected low frequency |
| Severe arrhythmias | Fisher’s exact test | RR (95% CI), RD | Expected rare |
| Cardiac arrest | Fisher’s exact test | RR (95% CI), RD | Expected rare |
| Any adverse event | Chi-squared or Fisher’s exact | RR (95% CI), RD | Composite endpoint |

Note: Fisher’s exact test is used when any expected cell count < 5 in the contingency table. Chi-squared test is used otherwise. Safety outcomes are analyzed descriptively; no multiple comparison adjustment is applied.

# 8. Subgroup Analyses

## 8.1 Pre-specified Subgroups

Interpretation of subgroup analyses will be treated with caution and considered exploratory rather than definitive. Subgroup analysis will be limited to the primary outcome (incidence of PSD on the first postoperative night) and will examine the following pre-specified subgroups: age (18–64 vs. ≥ 65 years), menopausal status (premenopausal vs. postmenopausal), postoperative sedative-hypnotic use (yes vs. no), postoperative pain intensity (NRS < 4 vs. ≥ 4), postoperative analgesic use (yes vs. no), PONV occurrence (yes vs. no), postoperative HADS-A scores (≥ 8 vs. < 8), postoperative HADS-D scores (≥ 8 vs. < 8), surgery duration (< 2 hours vs. ≥ 2 hours), time of return to ward (before 20:00 vs. after 20:00), and pathological diagnosis (benign vs. malignant).

## 8.2 Statistical Methods for Subgroup Analysis

| **Analysis Step** | **Method** | **Details** |
| --- | --- | --- |
| Interaction test | Wald test for interaction term(s) | Fit log-binomial (or modified Poisson) model including treatment × subgroup interaction |
| P-value adjustment | Benjamini–Hochberg correction | Applied to all interaction P-values to control FDR |
| Within-subgroup effects | Adjusted RR within each subgroup level | Estimate subgroup-specific aRR (95% CI) from the interaction model; interpret as exploratory |
| Heterogeneity assessment | Forest plot | Display subgroup-specific aRR (95% CI) and P-value for interaction |
| Interpretation | Conservative approach | Interpret subgroup effects cautiously when P-interaction ≥ 0.05 |

## 8.3 Forest Plot Specifications

Forest plots will be generated to display subgroup-specific treatment effects. Overall effect: diamond marker. Subgroup effects: square markers (size proportional to sample size). Reference line: vertical line at RR = 1.0. Display: adjusted RR (95% CI), BH-adjusted P-value for interaction.

# 9. Data Visualization Specifications

| **Figure Type** | **Application** | **Specifications** |
| --- | --- | --- |
| CONSORT flow diagram | Patient flow | Standard CONSORT format with enrollment, allocation to 3 arms, follow-up, analysis |
| Grouped bar chart | PSD incidence, PONV incidence by time point | Grouped bars for Control vs. Sham vs. Intervention; error bars: 95% CI; significance brackets |
| Forest plot | Subgroup analysis | Diamond for overall, squares for subgroups; size proportional to N; BH-adjusted P-interaction |
| Box plot | Continuous outcomes (AIS, QoR-15, HADS, NRS) | Median, IQR, whiskers to 1.5×IQR; individual points for outliers; 3 groups side-by-side |
| Line plot (spaghetti/mean) | Longitudinal outcomes over days 1–3 | Mean ± SE per group per time point; show group × time interaction |
| Stacked bar chart | Patient satisfaction distribution | Stacked proportions of Likert categories per group |

# 10. Summary of Statistical Methods by Outcome Type

| **Outcome Type** | **Examples** | **Normality Test** | **Unadjusted Test** | **Adjusted Analysis** | **Effect Measure** | **Multiple Comparison** |
| --- | --- | --- | --- | --- | --- | --- |
| Binary (Primary) | PSD night 1 | N/A | Chi-squared; log-binomial | Log-binomial or modified Poisson (robust SE) | aRR, RD (95% CI) | Bonferroni (P<0.025) |
| Binary (Secondary, repeated) | PSD nights 2–3, sedative use | N/A | Chi-squared or Fisher’s exact | GEE (logit, exchangeable) | aRR (95% CI) | BH (q<0.025) |
| Binary (Secondary, single) | PONV, analgesic use | N/A | Chi-squared or Fisher’s exact | Log-binomial or modified Poisson | aRR, RD (95% CI) | BH (q<0.025) |
| Continuous (repeated) | AIS, HADS, NRS, QoR-15 | Shapiro–Wilk | Mann–Whitney U | LMM (group × time + random intercept) | MD (95% CI) | BH (q<0.025) |
| Continuous (single) | PACU stay, hospital stay | Shapiro–Wilk | Mann–Whitney U or t-test | Linear regression | MD (95% CI) | BH (q<0.025) |
| Ordinal | Patient satisfaction | N/A | Kruskal–Wallis | Ordinal logistic regression | OR (95% CI) | BH (q<0.025) |
| Binary (Safety) | Adverse events | N/A | Fisher’s exact | Descriptive | RR (95% CI), P-value | None |

# 11. Abbreviations and Definitions

| **Abbreviation** | **Definition** |
| --- | --- |
| AIS | Athens Insomnia Scale |
| aRR | Adjusted Relative Risk |
| ASD | Absolute Standardized Difference |
| ASA | American Society of Anesthesiologists |
| BH | Benjamini–Hochberg (procedure) |
| BIS | Bispectral Index |
| BMI | Body Mass Index |
| CI | Confidence Interval |
| CONSORT | Consolidated Standards of Reporting Trials |
| FDR | False Discovery Rate |
| GEE | Generalized Estimating Equations |
| HADS-A | Hospital Anxiety and Depression Scale – Anxiety subscale |
| HADS-D | Hospital Anxiety and Depression Scale – Depression subscale |
| IQR | Interquartile Range |
| ITT | Intention-to-Treat |
| LMM | Linear Mixed-Effects Model |
| MAR | Missing at Random |
| MD | Mean Difference |
| MICE | Multiple Imputation by Chained Equations |
| NRS | Numerical Rating Scale |
| OR | Odds Ratio |
| PACU | Post-Anesthesia Care Unit |
| PONV | Postoperative Nausea and Vomiting |
| PP | Per-Protocol |
| PSD | Postoperative Sleep Disturbance |
| PSQI | Pittsburgh Sleep Quality Index |
| QoR-15 | Quality of Recovery-15 |
| RD | Risk Difference |
| RR | Relative Risk |
| SAP | Statistical Analysis Plan |
| SD | Standard Deviation |
| SE | Standard Error |
| SPIRIT | Standard Protocol Items: Recommendations for Interventional Trials |

# 12. Software Details

Statistical analyses will be performed using R software version 4.5.1 (R Foundation for Statistical Computing, Vienna, Austria; http://www.R-project.org). The following R packages will be utilized:

| **Package** | **Purpose** | **Key Functions** |
| --- | --- | --- |
| stats | Base statistical functions | chisq.test(), fisher.test(), t.test(), wilcox.test(), shapiro.test(), glm() |
| lme4 | Linear mixed-effects models | lmer(), glmer() |
| geepack | Generalized estimating equations | geeglm() |
| sandwich | Robust covariance estimation | vcovHC() |
| lmtest | Robust inference for models | coeftest() |
| emmeans | Estimated marginal means | emmeans(), contrast() |
| dplyr | Data manipulation | mutate(), filter(), select(), summarise() |
| tidyr | Data reshaping | pivot_longer(), pivot_wider() |
| ggplot2 | Figures | ggplot(), geom_bar(), geom_point(), theme_*() |
| forestplot | Forest plots | forestplot() |
| mice | Multiple imputation | mice(), with(), pool() |
| ordinal | Ordinal regression | clm() |
| tableone | Baseline characteristics table | CreateTableOne() |
| readxl | Read Excel files | read_excel() |
| openxlsx | Export formatted Excel tables | write.xlsx() |

# 13. References

1. Soldatos CR, Dikeos DG, Paparrigopoulos TJ. Athens Insomnia Scale: validation of an instrument based on ICD-10 criteria. J Psychosom Res. 2000;48(6):555–560.

2. Qiu D, Wang XM, Yang JJ, et al. Effect of Intraoperative Esketamine Infusion on Postoperative Sleep Disturbance After Gynecological Laparoscopy: A Randomized Clinical Trial. JAMA Netw Open. 2022;5(12):e2244514.

3. Buysse DJ, Reynolds CF 3rd, Monk TH, et al. The Pittsburgh Sleep Quality Index: a new instrument for psychiatric practice and research. Psychiatry Res. 1989;28(2):193–213.

4. Stark PA, Myles PS, Burke JA. Development and psychometric evaluation of a postoperative quality of recovery score: the QoR-15. Anesthesiology. 2013;118(6):1332–1340.

5. Hopewell S, Chan AW, Collins GS, et al. CONSORT 2025 statement: updated guideline for reporting randomised trials. BMJ. 2025;389:e081123.

6. Chan AW, Boutron I, Hopewell S, et al. SPIRIT 2025 statement: updated guideline for protocols of randomised trials. BMJ. 2025;389:e081477.

7. Naimi AI, Whitcomb BW. Estimating Risk Ratios and Risk Differences Using Regression. Am J Epidemiol. 2020;189(6):508–510.

8. Sawada M, et al. Choosing between log-binomial regression and modified Poisson regression for estimating risk ratios in epidemiologic studies. Am J Epidemiol. 2025.

9. Wason JMS, Robertson DS. Controlling type I error rates in multi-arm clinical trials: A case for the false discovery rate. Pharm Stat. 2021;20(1):109–116.

10. Schandelmaier S, Briel M, Varadhan R, et al. Development of the Instrument to assess the Credibility of Effect Modification Analyses (ICEMAN). CMAJ. 2020;192(32):E901–E906.

11. Fomenko A, et al. Hospital Anxiety and Depression Scale Anxiety subscale (HADS-A) for detecting anxiety disorders in adults. Cochrane Database Syst Rev. 2025;7(7):CD015456.

12. Gan TJ, et al. Consensus guidelines for the management of postoperative nausea and vomiting. Anesth Analg. 2014;118(1):85–113.
