# Supplementary material for: Intraoperative 40 Hz gamma frequency auditory stimulation for postoperative sleep disturbance in patients undergoing laparoscopic gynecological surgery: protocol for a randomized controlled trial
Source: Front Med (Lausanne). 2026 Apr 16;13:1813371. doi: 10.3389/fmed.2026.1813371 (PMC13128588; doi:10.3389/fmed.2026.1813371)
Supplement: Supplementary file 3 [file Supplementary_file_2.docx]

**Matlab code for 40 Hz sound**

% Parameters

fs = 44100; % Sample rate in Hz

dur = 1800; % Duration in seconds (= 30 minutes)

f = 40; % Sine wave frequency in Hz

amp = 0.999; % Amplitude (leave a little headroom to prevent clipping)

% Generate time axis and signal (double precision)

t = (0:1/fs:dur-1/fs).';

x = amp * sin(2*pi*f*t);

% 20 ms fade-in/fade-out to avoid transient clicks

fade = round(0.02*fs);

env = ones(size(x));

env(1:fade) = (0:fade-1).'/fade;

env(end-fade+1:end) = (fade-1:-1:0).'/fade;

x = x .* env;

% Write to WAV (16-bit PCM; change BitsPerSample to 32 for floating-point if needed)

audiowrite('40Hz_sound.wav', x, fs, 'BitsPerSample', 16);

% Optional: view file info

info = audioinfo('40Hz_sound.wav');

disp(info);
